# Supplementary material for: The interactome of intact mitochondria by cross-linking mass spectrometry provides evidence for coexisting respiratory supercomplexes
Source: Mol Cell Proteomics. 2017 Dec 8;17(2):216–32. doi: 10.1074/mcp.RA117.000470 (PMC5795388; doi:10.1074/mcp.RA117.000470)
Supplement: Supplemental Data [file supp_RA117.000470_133922_0_supp_23978_fzffwf.zip › spectra_annotation/mito_DR_spectra_annotation/212-1-1-1-3-1.pdf]

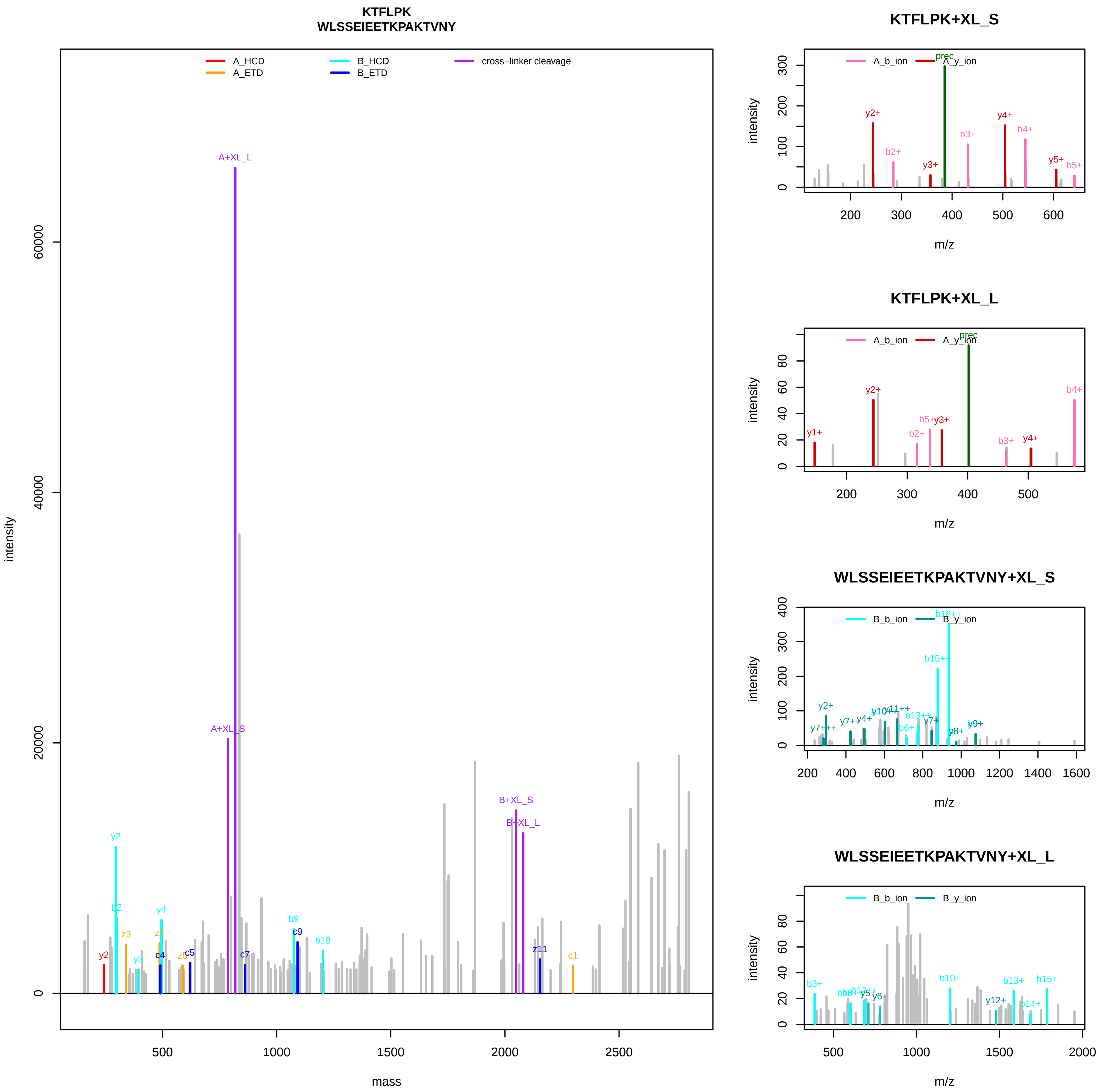
KTFLPK
WLSSEIEETKPAKTVNY
A_HCD
A_ETD
B_HCD
B_ETD
cross−linker cleavage
A+XL_L
A+XL_S
B+XL_S
B+XL_L
y2
b2
z3
y3
y4
z4
c4
z5
c5
c7
b9
c9
b10
z11
c1
intensity
mass
0
20000
40000
60000
500
1000
1500
2000
2500
KTFLPK+XL_S
A_b_ion
A_y_ion
prec
y2+
b2+
y3+
b3+
y4+
b4+
y5+
b5+
m/z
KTFLPK+XL_L
y1+
b5+
b3+
WLSSEIEETKPAKTVNY+XL_S
B_b_ion
B_y_ion
b16++
b15++
y10++
y11++
b13++
y7+++
y7++
b6+
y8+
y9+
WLSSEIEETKPAKTVNY+XL_L
b10+
b13+
b15+
y12+
b14+
y5+
y6+
b12++
b3+
